# β-TrCP Inhibition Reduces Prostate Cancer Cell Growth via Upregulation of the Aryl Hydrocarbon Receptor

**DOI:** 10.1371/journal.pone.0009060

**Published:** 2010-02-05

**Authors:** Udi Gluschnaider, Guy Hidas, Gady Cojocaru, Vladimir Yutkin, Yinon Ben-Neriah, Eli Pikarsky

**Affiliations:** 1 Department of Pathology and the Lautenberg Center for Immunology, Hebrew University Hadassah Medical School, Jerusalem, Israel; 2 Department of Urology, Hadassah-Hebrew University Medical Center, Jerusalem, Israel; Baylor College of Medicine, United States of America

## Abstract

**Background:**

Prostate cancer is a common and heterogeneous disease, where androgen receptor (AR) signaling plays a pivotal role in development and progression. The initial treatment for advanced prostate cancer is suppression of androgen signaling. Later on, essentially all patients develop an androgen independent stage which does not respond to anti hormonal treatment. Thus, alternative strategies targeting novel molecular mechanisms are required. β-TrCP is an E3 ligase that targets various substrates essential for many aspects of tumorigenesis.

**Methodology/Principal Findings:**

Here we show that β-TrCP depletion suppresses prostate cancer and identify a relevant growth control mechanism. shRNA targeted against β-TrCP reduced prostate cancer cell growth and cooperated with androgen ablation *in vitro* and *in vivo*. We found that β-TrCP inhibition leads to upregulation of the aryl hydrocarbon receptor (AhR) mediating the therapeutic effect. This phenomenon could be ligand independent, as the AhR ligand 2,3,7,8-Tetrachlorodibenzo-p-Dioxin (TCDD) did not alter prostate cancer cell growth. We detected high AhR expression and activation in basal cells and atrophic epithelial cells of human cancer bearing prostates. AhR expression and activation is also significantly higher in tumor cells compared to benign glandular epithelium.

**Conclusions/Significance:**

Together these observations suggest that AhR activation may be a cancer counteracting mechanism in the prostate. We maintain that combining β-TrCP inhibition with androgen ablation could benefit advanced prostate cancer patients.

## Introduction

Prostate cancer is the sixth most common cancer in the world and the third leading cause of cancer in men. In developed countries, prostate cancer develops in one of every nine men older than 65 years. This is a heterogeneous disease in terms of its clinical course and outcome but androgen signaling seems to be a common feature in its development and progression [1]. AR signaling is known to regulate normal, benign and cancerous prostate cells in various processes (e.g. differentiation and proliferation). In addition, there are observations indicating that AR expression is oncogenic in prostate epithelium and promotes progression to androgen independence [2]–[8]. The initial treatment of advanced stage and metastatic prostate cancer is suppression of androgen production by specific drugs or surgical castration. Later on, essentially all patients develop an androgen independent stage which does not respond to anti hormonal treatment. The latter phase is always lethal; thus, alternative strategies targeting novel molecular mechanisms are required.

The ubiquitin–proteasome system is a crucial determinant of virtually all biological processes in eukaryotes. In this pathway, proteins are targeted for degradation by covalent ligation to ubiquitin, a highly conserved small protein. Protein ubiquitination involves the concerted action of the E1 ubiquitin-activating enzyme, an E2 ubiquitin-conjugating enzyme and an E3 ubiquitin-protein ligase, the last of which delivers multiple ubiquitin molecules to the target protein [9]–[11]. Inhibiting the entire ubiquitin proteasome pathway has therapeutic value in cancer (e.g. Bortezomib,[12]). However, since proteasome inhibitors have pleiotropic effects, inhibition of a single key E3 may offer a more specific treatment option with fewer side effects.

Beta-transducin repeats-containing proteins (β-TrCP) serve as the substrate recognition subunits for the SCF^β-TrCP^ E3 ubiquitin ligases. These ligases ubiquitinate phosphorylated substrates specifically and play a pivotal role in the regulation of cell division and various signal transduction pathways, which in turn, are essential for many aspects of tumorigenesis [13]. There are two β-TrCP genes in mammalian genomes encoding for the two highly redundant proteins β-TrCP1 and β-TrCP2. In recent years, diverse β-TrCP substrates involved in different normal and malignant pathways were discovered [14]–[23]. Two well characterized *bona fide* substrates are β-catenin and IκB. Degradation of the latter frees NF-κB to enter the nucleus and induce transcription [11], [24]–[27]. On the other hand, β-catenin is phosphorylated, directly recognized by β-TrCP and degraded by the proteasome[24]. Thus, β-TrCP has opposing effects on two key oncogenic pathways. While the role of β-catenin in prostate cancer is controversial [28], [29], there is evidence suggesting that NF-κB plays a pro-tumorigenic role in prostate cancer [30]–[32]. The opposed effect on these two oncogenic proteins exemplifies the versatility of this particular E3 ligase. Furthermore, β-TrCP is directly implicated in several cancer types [33]–[36], thus it is plausible to further inquire its role in prostate cancer.

In this work, we show that β-TrCP inhibition inhibits prostate cancer growth showing additive effect with androgen ablation, *in vitro* and *in vivo*. This effect is largely mediated via activation of the aryl hydrocarbon receptor (AhR) as knocking down this protein abolishes the effect of β-TrCP inhibition.

## Results

### NF-κB Activation Correlates with Prostate Cancer Patients' Outcome

**Figure 1:**
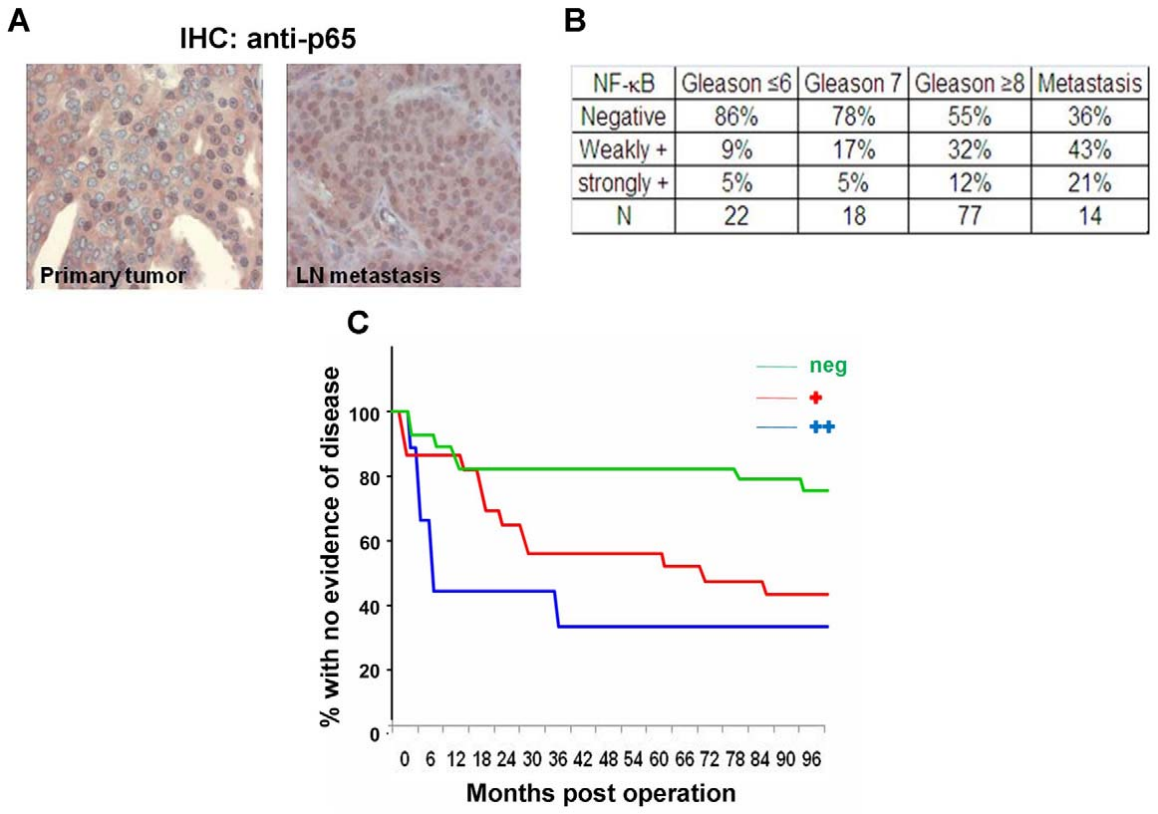
NF-κB activation correlates with prostate cancer patients' outcome. Prostate cancer sections were immunostained using p65 antibodies. A. Representative photomicrographs of a primary tumor (left) and lymph node metastasis (right) from the same patient. B. Percent of negative, positive and strongly positive cases spotted on a tissue microarray from the indicated Gleason scores (p = 0.0014, Fisher's exact test). C. primary prostate tumors (n = 131) were immunostained for p65 and assessed for NF-κB activity. Kaplan-Meier plot revealed a correlation between NF-κB status and the risk for disease recurrence. Blue, strongly stained samples; Red, weakly stained samples; Green, negative stained samples (p = 0.02).

β-TrCP is an important E3 ligase known to regulate many different substrates. NF-κB is a well characterized transcription factor negatively regulated by IκB, thus indirectly positively modulated by β-TrCP. The involvement of NF-κB in prostate cancer is well investigated and previous studies showed its tumorogenic role in the prostate. There is also recent indication for NF-κB involvement in the progression of prostate cancer to androgen independent stage [37]. In order to corroborate the relevance of this factor to prostate cancer progression, we tested its activation state in primary human prostate cancer samples using immunohistochemical staining with antibodies against p65 (**Figure 1**A). 131 prostate cancer specimens, spotted on a single tissue microarray slide from patients with different Gleason grades were analyzed. 39% of the samples showed at least some p65 positive nuclei (**Figure 1**B). We also analyzed 14 prostate cancer metastases, 64% of which were positive (**Figure 1**A, B). We set to evaluate the correlation between prostate cancer Gleason score and NF-κB activation. Indeed, we found an association between the two (p = 0.014, Fisher's exact test). It appeared that NF-κB nuclear staining was stronger in patients with higher Gleason score or in metastases raising the possibility that NF-κB activation could be correlated with prognosis. To test this possibility, we immunstained 80 primary tumors for p65, from patients that underwent radical prostatectomy and for which we had clinical follow-up. 77% of the patients whose primary tumor samples negatively stained for nuclear p65, showed no recurrence up to 8 years post radical prostatectomy. In contrast, the disease recurred in 65% of the NF-κB positive patients (**Figure 1**C). Another important pro-oncogenic β-TrCP substrate is β-catenin. Since β-TrCP inhibition would enhance β-catenin stability and promote tumorigenesis, it was necessary to monitor also β-catening correlation to prostate cancer patients' outcome. Nuclear localization of β-catenin activation did not correlate with disease severity in the same cohort of patients (Figure S1). Together, these results imply a clinical advantage for inhibiting β-TrCP in prostate cancer. Yet, the multitude of β-TrCP substrates (37, with possibly more to be identified) precludes a comprehensive analysis of all of them in the same way, and necessitates a direct approach to study the utility of β-TrCP inhibition in prostate cancer.

### β-TrCP Inhibition Reduces Prostate Cancer Cell Growth

**Figure 2:**
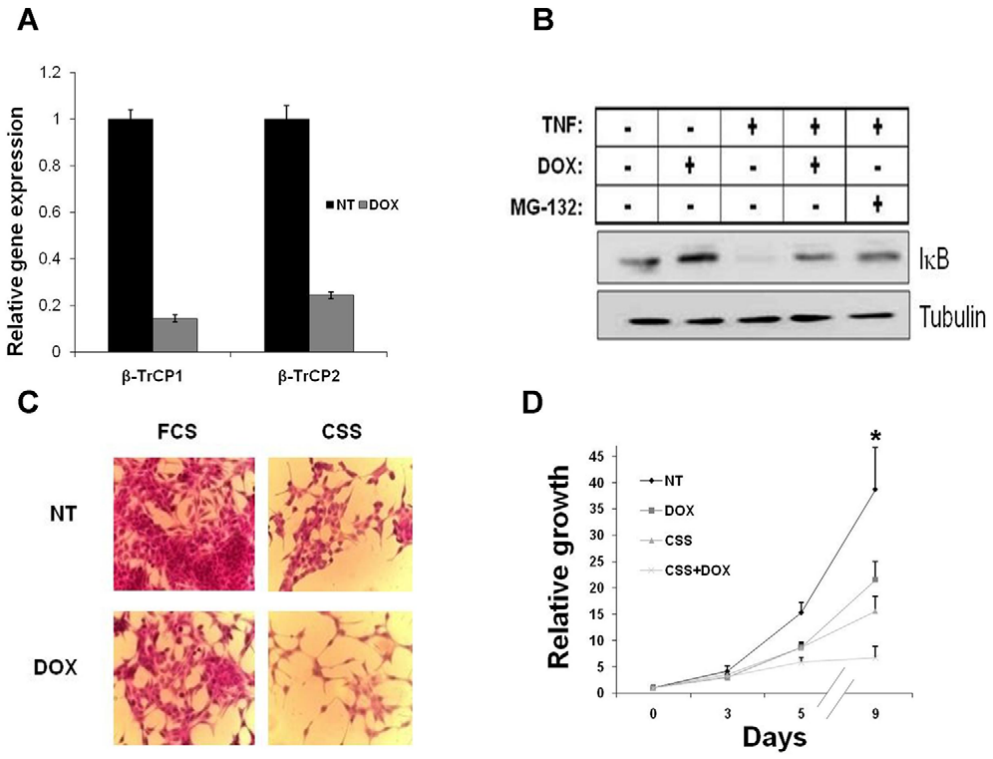
β-TrCP inhibition reduces prostate cancer cells growth. LNCaP cells were infected with a lentiviral vector bearing an inducible doxycycline dependent β-TrCP shRNA. A. Cells were treated for 72 hours with 1 µg/ml doxycycline and RNA levels of β-TrCP1 and β-TrCP2 were measured using qRT PCR. B. Western blot analysis of protein extracts from cells treated with TNF, doxycycline or MG-132 were immunoblotted with either IκB or tubulin. C. Representative photomicrograph of cells treated as indicated and stained with hematoxylin and eosin. D. XTT assay used to quantify cell growth at different time points. DOX, doxycycline; NT, no treatment; FCS, conditioned medium containing fetal calf serum; CSS, conditioned medium containing androgen depleted serum (charcoal stripped serum). Error bars, SD. * Significantly different from non treated cells and from each other, p-value<0.01, t-test.

We next examined the effect of β-TrCP inhibition on prostate cancer cell growth. To this end, we constructed a doxycycline inducible shRNA lentiviral vector targeting both β-TrCP1 and β-TrCP2 with which we transduced the two androgen-sensitive human prostate cancer cell lines, LNCaP and LAPC4. Doxycycline treatment of the trasnduced LNCaP cells resulted in efficient knock down of both β-TrCP genes (85% and 75% for β-TrCP1 and β-TrCP2, respectively) as determined using quantitative real time PCR (qRT PCR - **Figure 2**A). This was accompanied by IκBα stabilization both before and following TNF stimulation, demonstrating efficient induction of protein down regulation (**Figure 2**B). To evaluate the consequence of β-TrCP knock down on prostate cancer cells, we monitored doxycycline-treated and untreated LNCaP cells for nine days and measured cell growth *in vitro*. **Figure 2**C shows diminished cell growth after either β-TrCP inhibition or androgen ablation treatment (lower left and upper right panels, respectively). Moreover, combining β-TrCP inhibition with androgen ablation shows an additive effect (**Figure 2**C lower right panel and **Figure 2**D). We next quantified cell growth using the XTT method and confirmed our morphologic analysis (**Figure 2**D). Similar results were obtained with LAPC4 cells (Figure S2).

To rule out shRNA off target effects, we used a previously described β-TrCP dominant negative construct lacking the F-box domain [33]. This variant stabilizes β-TrCP substrates as it binds the phosphorylated targets, but fails to recruit the additional components of the SCF complex which are critical for E3 ligase catalytic activity. The β-TrCP inhibition effects using the dominant negative form, were essentially similar to the shRNA results (Figure S3A and B).

### β-TrCP Inhibition Cooperates with Androgen Ablation *In Vivo*

**Figure 3:**
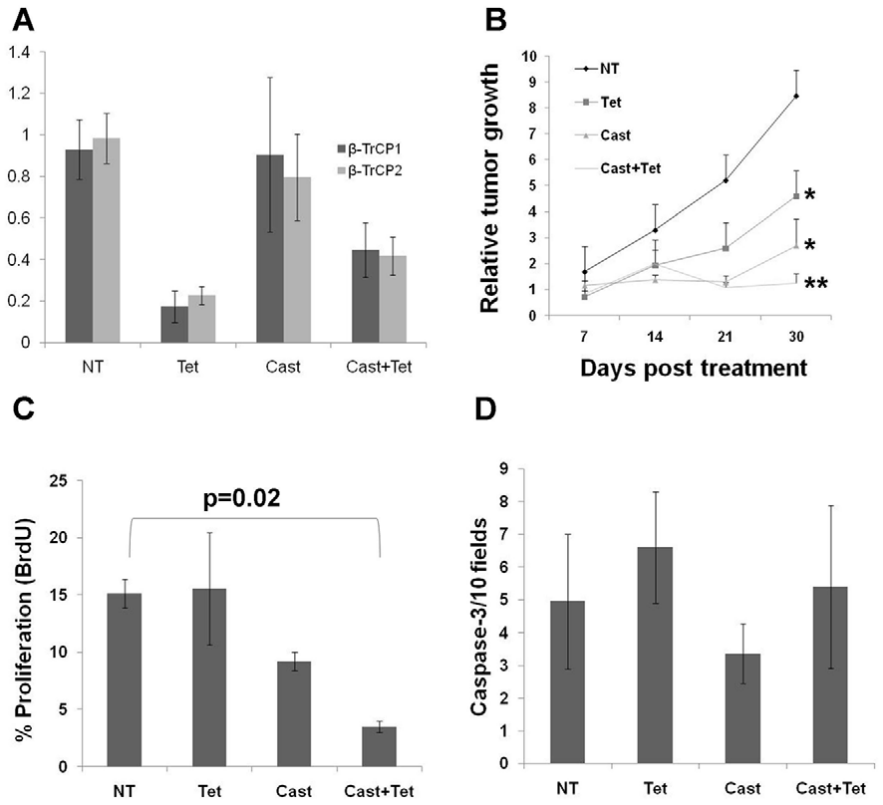
β-TrCP inhibition cooperates with androgen ablation treatment *in vivo*. LNCaP cells bearing a tetracycline induced β-TrCP shRNA construct were injected subcutaneously to immunosuppressed *Rag1* ^−/−^ mice. Mice (n≥4 in each group) were either untreated (NT), treated with tetracycline in their drinking water (Tet), physically castrated (Cast) or both (Cast+Tet) for 30 days. Tumor volumes were measured weekly. (A). qRT PCR for both β-TrCP isoforms was performed on RNA extracted from the tumors harvested at day 30. (B) Tumor growth kinetics. Tissue sections were stained for BrdU (C) or activated caspase 3 (D) and the proliferation and apoptosis scores, respectively, were determined for each tumor. Shown are mean ± standard deviation (A) or ±S.E.M (B, C and D).* p-value<0.05, ** p-value = 0.0002, t-test; p-value in C refers to t-test.

To further analyze the effect of β-TrCP inhibition on human prostate cancer tumor growth, we used a xenograft model. We injected LNCaP cells carrying an inducible β-TrCP shRNA construct into the subcutis of 39 immunosuppressed *Balb/c Rag1* ^−/−^ male mice. 25 of the mice (64%) developed visible and measurable tumors 30 days post injection. Since initial tumor development was somewhat heterogeneous in size and kinetics, we randomly divided these 25 mice into four treatment groups: 1. Intact untreated mice (NT, n = 4; mean tumor volume 123.1±70.7); 2. Mice given tetracycline in the drinking water resulting in β-TrCP inhibition (Tet, n = 5; mean tumor volume 80.3±98.2); 3. Castrated mice resulting in androgen ablation (Cast, n = 7; mean tumor volume 121.6±95.0); 4. Castration plus tetracycline (Cast + Tet, n = 8; mean tumor volume 180.3±143.9). There was no significant difference in average tumor size at initiation of treatment between the four groups. We monitored tumor growth once a week, and sacrificed the mice 30 days post the initial treatment. RNA extracted from tumors from the Tet treated mice showed β-TrCP knock down ranging from 50% to 80% (**Figure 3**A). Similar to our *in vitro* findings, inhibiting β-TrCP reduced tumor growth with or without androgen ablation (**Figure 3**B). Analysis of tumor proliferation using BrdU immunostaining revealed that the mice treated with both androgen ablation and β-TrCP inhibition showed the lowest proliferation rates (**Figure 3**C and Figure S4). Tumor growth suppression could not be explained via apoptosis, since anti cleaved caspase-3 immunostaining did not reveal any differences between treatment groups (**Figure 3**D). Similar results were obtained with AT2.1 rat prostate cancer cells stably transfected with an inducible dominant negative β-TrCP transgene (Figure S2B). In conclusion, our results indicate that β-TrCP inhibition suppresses prostate cancer growth both *in vitro* and *in vivo* and shows an additive effect with androgen ablation.

### Aryl Hydrocarbon Receptor (AhR) Is Upregulated upon β-TrCP Inhibition and Androgen Ablation

**Figure 4:**
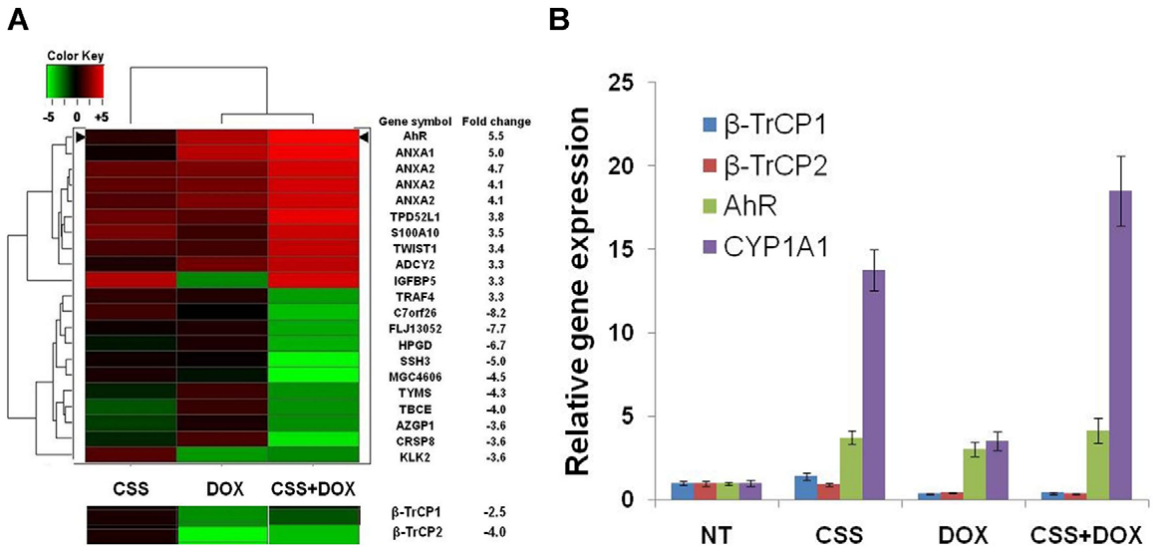
Aryl hydrocarbon receptor (AhR) expression is increased after androgen ablation and β-TrCP treatments. A. LAPC4 cells infected with inducible β-TrCP shRNA were treated for 72 hours with the indicated treatment, subjected to RNA extraction and cDNA microarray analysis (Affymetrix). After data normalization, gene expression profiles were compared between treatment and the untreated control samples. A. Heat map dendrogram showing the ten most highly up (red) and down (green) regulated genes due to the combined treatment. Fold change refers to the combined treatment probes values relative to control. Expression of β-TrCP isoforms is presented below. B. LAPC4 cells infected with the same lentiviral vector and treated as indicated were subjected to RNA extraction and qRT PCR analysis with the listed primers. CSS, charcoal stripped serum; DOX, doxycycline; Error bars, SD.

To elucidate the molecular pathways that are responsible for the growth inhibitory effect of β-TrCP depletion in combination with androgen ablation we conducted a wide range microarray analysis. LAPC4 cells infected with the shβ-TrCP lentiviral vector, were either left untreated or treated for 72 hours with doxycycline, charcoal stripped serum or both. cDNA samples were subjected to microarray analysis using U133 Affimetrix chips probing ∼30,000 probe sets. We then sought to identify genes which are cooperatively affected by both androgen ablation and β-TrCP inhibition. Among the upregulated genes, were potential anti inflammatory genes (e.g. ANXA1) and among the prominently downregulated were prostate specific genes (e.g. KLK2). Yet, the most dramatic increase was the expression of the aryl hydrocarbon (dioxin) receptor (AhR). This gene was upregulated upon either androgen ablation or β-TrCP inhibition and was the most highly changed gene due to the combined treatment (**Figure 4**). We could attribute AhR level upregulation to β-TrCP depletion, since doxycycline alone did not alter the receptor's mRNA (Figure S5).

**Figure 5:**
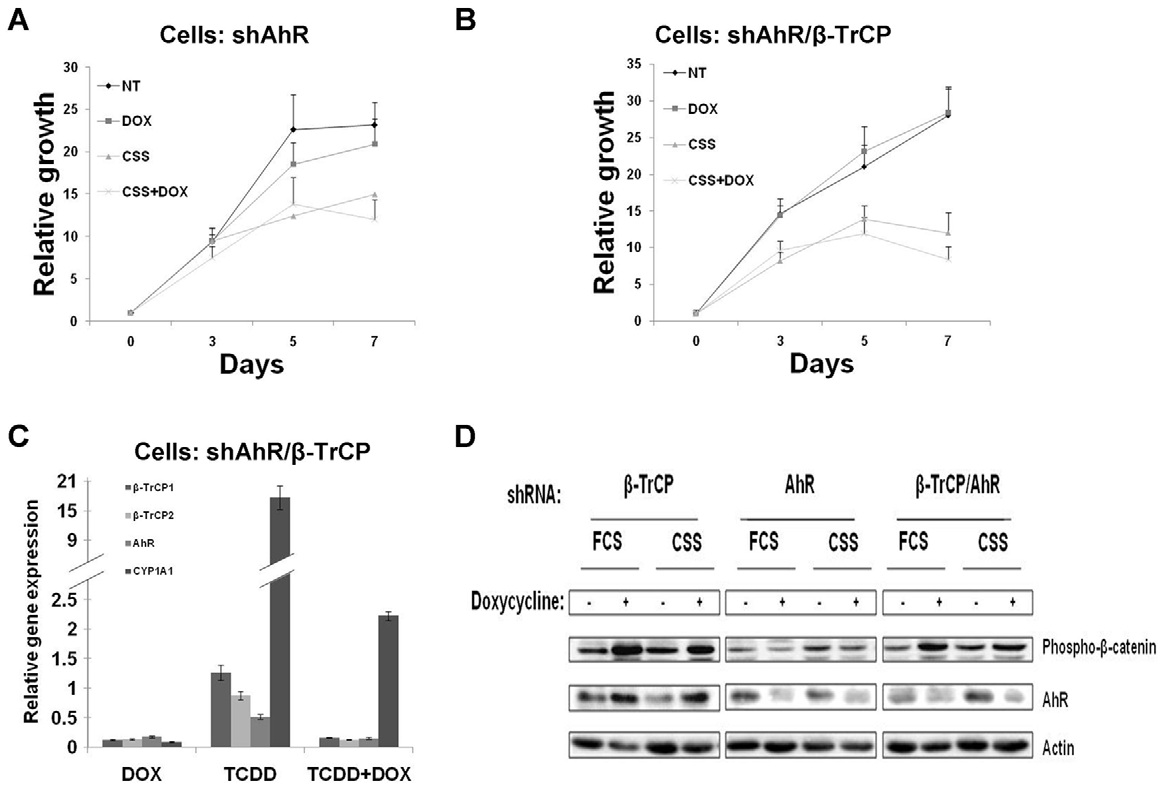
AhR mediates the β-TrCP inhibition phenotype. LNCaP cells infected with an inducible lentiviral vector for shAhR alone (A) or together with shβ-TrCP (B) were treated for 72 hours with the indicated treatments and cell growth was measured by the XTT assay. C. qRT PCR confirmed β-TrCP and AhR efficient knock down. D. Western blots showing β-catenin stabilization after β-TrCP inhibition and confirming AhR protein level elevation or knock down due to relevant shRNAs. DOX, doxycycline; FCS, fetal calf serum; CSS, charcoal stripped serum. Error bars, SD.

The AhR is a ligand activated transcription factor involved in organogenesis, in detoxification of endo- and xenobiotics and in mediating diverse organ-specific toxic responses of dioxins. This receptor belongs to the basic helix- loop-helix (bHLH)/PAS (Period -Aryl hydrocarbon receptor nuclear translocator-Single minded) family of heterodimeric transcriptional regulators. bHLH/PAS proteins are involved in the control of diverse physiological processes such as circadian rhythms, organ development, neurogenesis, metabolism and the stress response to hypoxia [38]–[40]. Recent studies revealed a connection between the AhR pathway and prostate cancer both *in vitro* and *in vivo*, showing that the AhR interacts and inhibits the AR. Moreover, the AhR acts as an E3 ligase of the AR [41] and AhR *null* TRAMP mice show increased prostate tumorigenesis [42]. We used qRT PCR to validate the cDNA array analysis. We found that while each treatment alone increased AhR RNA levels more than 2 folds, the combined treatment resulted in more than 4 fold upregulation in both LAPC4 (**Figure 4**B) and LNCaP cells (Figure S6). To test the functional activity of the AhR pathway we measured the mRNA levels of its canonical target cytochrome p450 1A1 (CYP1A1). Our analyses show that CYP1A1 levels were increased in correlation with AhR levels in the 4 treatment groups (Figure 4B). It should be noted that this upregulation occurred without addition of an exogenous AhR ligand. Addition of the potent AhR ligand TCDD further augmented CYP1A1 upregulation (**Figure 5**C and data not shown).

### The Growth Suppression Effect of β-TrCP Inhibition Is Mediated via Upregulation of the Aryl Hydrocarbon Receptor

To investigate the significance of the AhR pathway activation following β-TrCP depletion, we first infected LNCaP cells with inducible AhR shRNA. While doxycycline treatment resulted in reduced AhR mRNA levels we could not detect any effect on cell growth (**Figure 5**A). Next we co-infected LNCaP cells bearing inducible β-TrCP shRNA with the inducible AhR shRNA vector. Addition of doxycycline to the medium of double knockdown cells resulted in reduced β-TrCP mRNA levels, similar to the single knockdown cells; yet as expected, in these cells AhR levels were decreased rather than increased both at the mRNA and protein levels (**Figure 5**C, D). Thus, the double knockdown cells allow us to test whether the growth inhibitory effect of β-TrCP modulation is mediated via AhR upregulation. Indeed, in the double knockdown LNCaP cells, β-TrCP depletion failed to reduce cell growth either with or without androgen ablation (**Figure 5**B). Similar results were obtained with a different shRNA targeted against the AhR (data not shown). Interestingly, addition of the potent exogenous ligand TCDD did not reduce cell growth alone; nor had it an effect with any of the different treatments (Figure S7). This suggests that this AhR effect is ligand independent. Western blot analysis confirmed β-catenin stabilization and the AhR mRNA upregulation upon β-TrCP inhibition (**Figure 5**C). Thus, the double knockdown results indicate that most of the effect of β-TrCP knockdown is mediated by upregulation of the AhR.

### AhR Expression in Prostate Cancer Patients

**Figure 6:**
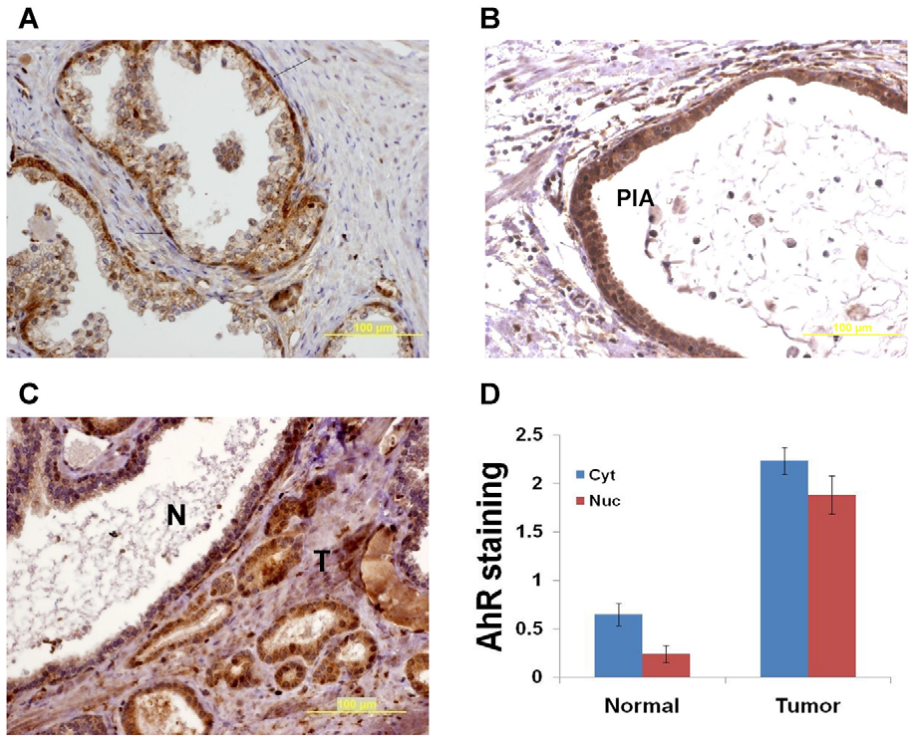
AhR is upregulated in malignant prostate cells. Prostate sections were immunostained with AhR antibody. Photomicrographs show strong AhR expression in basal cells (arrows in A) and proliferative inflammatory atrophy (B). C. Higher AhR expression is noted in malignant glands (T) compared with normal glands (N). D. Normal and malignant glands were scored using a 0–3 scale. Mean values ±S.E.M. are shown (p-value<0.001, Mann-Whitney test).

Observing AhR upregulation upon β-TrCP inhibition in prostate cancer cells prompted us to inspect AhR status in various stages of prostate cancer. To address this aim, we collected 39 specimens of primary prostate cancer tumors from the Hadassah Medical Center. Out of this cohort, 17 patients suffered from disease recurrence. We performed immunohitochemical anti AhR staining and monitored cytoplasmatic and nuclear AhR expression. First, we detected high AhR in basal cells located in the benign gland perimeter (**Figure 6**A). Interestingly, proliferative inflammatory atrophy (PIA), considered as a precursor lesion to prostate cancer showed very strong cytoplasmic and nuclear staining (**Figure 6**B). We used a subjective score from 0 to 3 to quantitate staining intensity in normal and malignant epithelial cells in each specimen. Our analysis demonstrates upregulation of AhR in both the cytoplasmic and nuclear locations in the malignant epithelium (**Figure 6**C, D, p<0.001, Mann-Whitney test). However, we did not observe a correlation between AhR expression in the tumor cells and disease recurrence (data not shown).

## Discussion

Prostate cancer is a heterogeneous disease comprising many genetic and phenotypic features. One important hallmark of prostate cancer is progression to an androgen independent stage (AI) after hormonal treatment. The causes for this transition are not fully understood and adjuvant treatments fortifying hormonal manipulation are required. One factor that is often implicated in tumor progression in many types of cancer is NF-κB. Here we confirm that NF-κB is often activated in advanced prostate cancer patients (**Figure 1**). Moreover, NF-κB activation correlates with prostate cancer recurrence (**Figure 1**C). One of the major regulators of NF-κB is the E3 ubiquitin ligase SCF^β-TrCP^, which targets IκB as well as many other substrates for ubiquitination and degradation, [27]. β-TrCP is thought to play a pro-tumorigenic role in certain types of cancers [43]. Based on observed upregulation of NF-κB, it was important to follow another common pro-oncogenic β-TrCP substrate, β-catenin, which was also implicated in prostate cancer [28], [29], [44]. However, we could not detect a correlation between β-catenin activation and prostate cancer recurrence (Figure S1), suggesting that only some β-TrCP targets are of relevance to the disease progression. To evaluate the full scope of β-TrCP effects, we set out to inhibit β-TrCP in prostate cancer cells and to monitor its effect on prostate cancer cell growth. Our *in vitro* and *in vivo* studies revealed that upon β-TrCP inhibition prostate cancer cell growth is reduced. We could also detect an additive effect when combining β-TrCP inhibition with androgen ablation (**Figure 2** and 3). To elucidate the mechanisms of growth suppression by β-TrCP inhibition, we carried a cDNA array analysis and observed an additive upregulation of the AhR (**Figure 4**). AhR was previously demonstrated to act as an E3 ligase of the AR [41], providing us a possible link to AR signaling. Moreover, we noted that AhR expression is higher upon combining androgen ablation and β-TrCP inhibition. We therefore hypothesized that the upregulation of the AhR could be mediating the growth inhibitory effect of β-TrCP knockdown in prostate cancer cells. This notion was supported by previous reports demonstrating an inhibitory role for the AhR pathway in prostate cancer. Morrow *et al* showed an AhR ligand dependent growth inhibition in LNCaP cells. Likewise, other studies have also implicated the androgen receptor in AhR growth inhibition [45], [46]. In our study, knocking down the AhR *per se* in LNCaP cells did not alter prostate cancer cell growth (**Figure 5**A), suggesting that the basal levels of AhR do not exert an inhibitory effect unless stimulated by ligand. On the other hand boosting AhR expression via β-TrCP depletion was associated with considerable ligand-independent growth suppression. AhR depletion reversed the growth suppression effect of β-TrCP knockdown, even under androgen ablation, proving that AhR upregulation, which we also noted in other stress conditions (e.g. atrophy, inflammation and androgen ablation) accounts for the β-TrCP inhibitory effect (**Figure 5**). Interestingly, ligand administration did not affect cell growth even though it did upregulate expression of the classic AhR pathway target CYP1A1. These results implicate a ligand and CYP1A1 independent AhR pathway in prostate cancer cells.

Chesire *et al* identified the AhR as a putative β-catenin target in LNCaP cells [47]. As we show that β-TrCP depletion stabilizes β-catenin along with the upregulation of AhR (**Figure 5**D), it is possible that the cause of AhR elevation following β-TrCP depletion is β-catenin stabilization.

Our studies indicate a novel ligand independent strategy of boosting AhR expression as means of suppressing prostate cancer growth. This strategy may also be echoed in the natural history of prostate cancer. We found that AhR is normally expressed at low levels in the prostatic epithelium basal cells (cytoplasmic and nuclear staining, **Figure 6**A) and is substantially upregulated in areas of proliferative inflammatory atrophy (PIA), considered a precursor lesion to cancer (**Figure 6**B). AhR activation in basal and atrophic cells may therefore be viewed as an anticancer mechanism. It is possible that microenvironmental inflammatory signals are responsible for such stress induced signaling. A mutation in one allele of β-TrCP1 was identified in one human prostate tumor in a systematic screen of Wnt pathway mutations [48]. However this mutation is unlikely to have an effect on the NF-κB pathway as the other allele and possibly both β-TrCP2 alleles were wild type. Similarly, to our knowledge, activating mutations in the NF-κB pathway were so far not reported in human prostate tumors. Nevertheless, NF-κB known to mediate malignant transformation is constitutively upregulated in this tumor type via multiple mechanisms. We conclude that different stress signals, including inflammation, atrophy and androgen ablation upregulate AhR expression in both normal and malignant prostate cells and conduct a protective mechanism. Inhibiting β-TrCP at advanced disease stages may be relevant in developing strategies for enhancing the efficacy of prostate cancer treatments.

## Materials and Methods

### Ethics Statement

Experiments with human tissues were approved by Institutional Review Board, Hadassah-Hebrew University Medical Center. Due to the retrospective nature of this study and according to the declaration of Helsinki, participants were not obtained constantly informed. In addition, our IRB waived the need for written informed consent. All mice experiments were approved by the IACUC.

### Dominant Negative β-TrCP (WD)

*Dominant negative β-TrCP (WD)* was cloned using E3RS excluded from pCDNA3-EE-hE3RS plasmids with the primers 5′ to 3′ forward: GCGGCCGCTATGGACCC-GGCCGAG (with *NotI* site in its 5′); reverse: TTATCTGGAGATGTAGGTGT; the product was cloned into TA vector (Invitrogen). The last vector was cut with *AvrII/ASP718*, filled in and blunt ligated. The relevant fragment was cut and inserted into pFLAG-CMV™-2 expression vector (Sigma-Aldrich) with *NotI/BamHI*. This procedure produced a WD construct lacking part of the F-box and conjugated to FLAG. The WD-FLAG was inserted under bidirectional teracycline promoter expressing GFP. The resulting plasmid was transfected into AT2.1 Rat prostate cancer cells expressing the tetracycline trans-activator, using FUGENE reagent (Roche Applied Science). The transfected cells were selected using hygromycin and neomycin (Sigma-Aldrich) containing media to produce a stable clone which expresses a dominant negative β-TrCP upon addition of tetracycline or its derivate doxycycline.

### Inducible β-TrCP and AhR shRNA

The human shRNA 5′- GUGGAAUUUGUGGAACAUC 3′ targeted against β-TrCP1 and β-TrCP2 was constructed into pTER plasmid and inserted into a modified pRRL.sin.PPT.tetO7.MCS.PRE lentiviral vector. The vector consists of an HI promoter, tet operator, the shRNA coding sequence and eF1α promoter driving the tet repressor fused to eGFP. Virus production and infection were carried out as previously described [49]. To target the AhR we used the following shRNA sequences: 1. 5′ CAGCUGAAUUAAAUAACAU 3′; 2. 5′ CAGACAGUAGUCUGUUAUA 3′. Both of which proved to be efficient in knocking down the receptor (the presented data represent those obtain with the latter sequence). shRNA expression is induced only after Tetracycline or Doxycycline (Sigma-Aldrich) administration. The vector alone was used as control. Double knocked down LNCaP cells were designed by co-infecting shβ-TrCP cells with lentiviral vectors carrying either of the above described sequence.

### Cell Culture

DMEM, RPMI 1640, Trypsin EDTA, Penicillin-Streptomycin solution, L-glutamine, fetal calf serum (FCS), Charcoal Stripped Serum (CSS) were purchased from Biological Industries, Kibbutz Beit Haemek, Israel. LAPC4 cells were kindly provided by Prof. Zelig Eschar (The Weizmann Institute of Science, Rehovot, Israel); AT2.1, LNCaP and 293T were furnished to us by Dr. Rachel Bar-Shavit (Department of Oncology, Hadassah Medical Center, Jerusalem, Israel). All cell lines were incubated at 37°C 5% CO_2_ in appropriate medium containing 10% FCS or CSS as indicated.100 pM Methyltrienolone (R1881, Perkin-Elmer, New England Nuclear) was added to LAPC4 full media.

### Cell Proliferation Assay

MTT (3-(4,5-Dimethylthiazol-2-yl)-2,5-diphenylte-trazolium bromide) and XTT (2,3-Bis(2-methoxy-4-nitro-5- sulfophenyl)-2H-tetrazolium-5-carboxanilide) was used as the protocol indicates (Biological Industries, Kibbutz Beit Haemek, Israel). All experiments were carried out in 96 well plates with eight repeats of at least 3 independent infections.

### Western Blotting

Whole-cell lysates were prepared from transfected or infected cells by extraction in lysis buffer containing 50 mM Tris (pH 8), 150 mM NaCl, 1% NP-40, 0.1% SDS, 10 mM NaF, 1 mM Na3VO4, 1 mM phenylmethylsulfonyl fluoride, 1 mg/ml leupeptin, 1 mg/ml aprotinin and 1 mM dithiothreitol. Proteins were resolved by 10% SDS-PAGE, transferred onto nitrocellulose membranes, probed with appropriate antibodies, incubated with Peroxidase-conjugated Goat anti mouse or Rabbit IgG (Jackson Laboratories) and developed using the ECL kit (Pierce). Primary antibodies used were: anti-Flag (Sigma); anti-IkB, anti-phpspho-IkB (Cell Signaling); Aryl Hydrocarbon Receptor, CYP1A1 (Santa-Cruz); phospho-β-catenin (BD Biosciences).

### Xenografts

AT2.1 or LNCaP cells were harvested washed and reconstituted in PBS. 10^6^ 51B cells per 200 µl volume were injected subcutaneously to 6–7 weeks old atymic (*Nude*) male mice. 10^6^ LNCaP cells were injected to 6–7 weeks old *rag1−/−* male mice together with Matrigel (BD bioscience). Tumors were measured in two dimensions with caliper, and tumor volume (mm^3^) was calculated with the formula V = (lengthXwidth^2^)/2. Half of the mice received Doxycycline (0.2 mg/ml, AT2.1) or tetracycline (1.5 mg/ml, LNCaP) supplemented with 5% sucrose in their drinking water. Half of the mice were surgically castrated: mice were anesthetized using Ketamine/2% Xylazine at 5.7∶1 ratio (0.1 ml per 25–30 gram mouse). Surgical castration was performed via a midline scrotal incision allowing bilateral access to the hemiscrotal contents. After exposing each testicle, a 3-0 Vicryl suture was used to ligate the spermatic cord and then remove the testicle. Mice were treated with Carprofen (Rimadyl) as analgesics after surgery. Two hours before sacrifice, mice were injected with BrdU intraperitoneally 100 µl per 10 grams of body weight (RPN201, Amersham Pharmacia Biotech Inc). For AT2.1 xenografts, *NUDE* mice were treated pre-injection and tumors were weighted at the end of the experiment. LNCaP xenografted mice were treated 30 days post injection when measurable tumors were established. Tumor relative growth was calculated individually for each mouse, comparing each week's measurement to the treatments' day 0 (30 days post injection). All mice experiments were approved by the IACUC.

### Immunohistochimestry

mouse tumor specimens were fixed in 4% neutral-buffered formalin and embedded in paraffin. Patients paraffin embedded samples were collected from the archives of the Department of Pathology at the Hadassah-Hebrew University Medical Center. Experiments with human tissues were approved by the institutional review board. 5 µM sections were dewaxed and hydrated through graded ethanol dilutions, then cooked in appropriate buffer (pH 7.4) in a pressure cooker at 115°C for 3 minutes. Endogenous peroxidase activity was blocked with 3% hydrogen peroxide followed by washing. The sections were then incubated with the indicated antibodies: anti-p65 (Neomarkers; 1∶100), anti-β-catenin (Santa Cruz; 1∶300) and anti-AhR (Santa Cruz; 1∶200). All sections were counterstained with hematoxylin.

### RNA, cDNA Micrroarray and Real-Time PCR

Total RNA was extracted from LAPC4 or LNCaP cells infected with β-TrCP shRNA lentiviral vector with TRI Reagent (Sigma). For cDNA microarray RNA was extracted from LAPC4 infected cells using TRIzol® (Invitrogen). cRNA preparation and hybridization was performed using standard manufacture protocol; Biotin-labeled target synthesis reactions were performed using standard protocols supplied by the manufacturer (Affymetrix, Santa Clara CA, USA). From each RNA sample, 5 µg were converted into double-stranded cDNA by reverse transcription with SuperScript™ II Reverse Transcriptase (Life Technologies, Helgerman CT, USA), using T7-oligo-dT as a primer. Expression value (signal) was calculated using Affymetrix Genechip software MicroArray Suite 5.0. Only probe sets that had at least an intensity of 20 and a present call at one of the microarrays were selected. Next quantile normalization was applied to the log2 transformed expression values (Bolstad BM Bioinformatics 19: 185–193). For β-TrCP knockdown determination and validation studies 2 µg of RNA were used as template for synthesis of cDNA using SuperScript™ II Reverse Transcriptase. The cDNA was subsequently used as Real Time PCR template. All Real Time PCR reactions were carried out using Absolute Blue QPCR SYBR Green Low ROX Mix (ABgene) with the following primers (5′ to 3′): β-TrCP1 Forward: ATCGGATTCCACGGTCAGAG, Reverse: AATCAACGTGTTTAGCATT-TCACCT; β-TrCP2 Forward: CCATCAAAGTCTGGAGCACGA, Reverse: CGCT-TGTGCCCATTGAGAGTA; AhR Forward: ACATCACCTACGCCAGTCG, Reverse: CTCTATGCCGCTTGGAAGGAT; CYP1A1 forward: TGAATGCCTTCAAGGAC-CTG, Reverse: TCAGGCTGTCTGTGATGTCC.

All microarray data is MIAME compliant. The raw data has been deposited in GEO (accession number GSE19141).

## Supporting Information

Figure S1 β-catenin activation does not correlate with prostate cancer patients' outcome. Primary prostate cancer tumors were immunostained using β-catenin antibodies. A. Representative photomicrographs of samples from negative (left) and positive (right) β-catenin stained tumors. B. Kaplan Meier curves plotting β-catenin positive (blue) vs. negative (green) patients' recurrence free interval. Scale bars in A, 50 µM.
(1.02 MB TIF)

Figure S2 β-TrCP shRNA cooperates with androgen ablation to reduce LAPC4 cell growth. LAPC4 cell infected with the mentioned inducible lentiviral vector containing β-TrCP shRNA. A. qRT-PCR demonstrating efficient β-TrCP1 and β-TrCP2 knockdown. B. XTT assay was used to quantify cells proliferation rates (means ± S.E.M.). Error bars in A, SD. NT, no treatment; DOX, doxycycline; CSS, charcoal stripped serum. * All treatments were statistically different from control (p-value<0.05, t-test).
(0.19 MB TIF)

Figure S3 Dominant negative β-TrCP expression inhibits prostate cancer cell growth in vitro and in vivo. A. AT2.1 cells stably transfected with an inducible dominant negative β-TrCP were treated as indicated for 72 hours and subjected to MTT cell proliferation assay. B. LNCaP cells infected with lentiviral vector expressing eGFP (GFP) or dominant negative β-TrCP (ΔF-box) and assayed using the XTT reagent. C. Athymic 6–8 weeks male NUDE mice were divided into the 4 indicated groups (n≥4) and subcutaneously grafted with AT2.1 cells bearing the doxycycline dependent dominant negative β-TrCP construct. Tumor volumes were measured two weeks post injection. Shown are means ± S.E.M for A and C and means ± SD for B. * Significantly different from control group (p<0.05, t-test); ** Significantly different from all treatment groups (p<0.01, t-test). NT, no treatment; DOX, doxycycline; CSS, charcoal stripped serum; Cast, castrated mice.
(0.20 MB TIF)

Figure S4 β-TrCP inhibition cooperates with androgen ablation treatment to reduce prostate cancer cells proliferation in vivo. LNCaP xenografts from treated Rag1^−/−^ mice were immunostained with anti BrdU antibodies. Representative photomicrographs for each of the four treatment groups are shown. NT, no treatment; cast, castrated mice; Tet, tetracycline.
(2.87 MB TIF)

Figure S5 Doxycycline does not upregulate AhR. LNCaP cells infected with a GFP expressing lentiviral vector were subjected to qRT PCR analysis with the indicated primers. Means ± S.E.M of the relative genes expressions are shown. NT, no treatment; DOX, doxycycline.
(0.12 MB TIF)

Figure S6 β-TrCP inhibition upregulates the AhR in LNCaP cells. LNCaP cells infected with an inducible shβ-TrCP lentiviral vector and treated as indicated were subjected to RNA extraction and qRT PCR analysis with the listed primers. CSS, charcoal stripped serum; DOX, doxycycline; Error bars, SD.
(0.33 MB TIF)

Figure S7 TCDD does not alter LNCaP cell growth in vitro. LNCaP cells were infected with a lentiviral vector harboring an inducible doxycycline dependent β-TrCP shRNA. Cells were treated with 1 µg/ml doxycycline, 10 nM TCDD or both and XTT assay was used to quantify cell growth at different time points. Shown are means ± SEM. NT, no treatment; DOX, doxycycline.
(0.07 MB TIF)
