# Supplementary figures and images for: β-TrCP Inhibition Reduces Prostate Cancer Cell Growth via Upregulation of the Aryl Hydrocarbon Receptor
Source: PLoS One. 2010 Feb 5;5(2):e9060. doi: 10.1371/journal.pone.0009060 (PMC2816705; doi:10.1371/journal.pone.0009060)

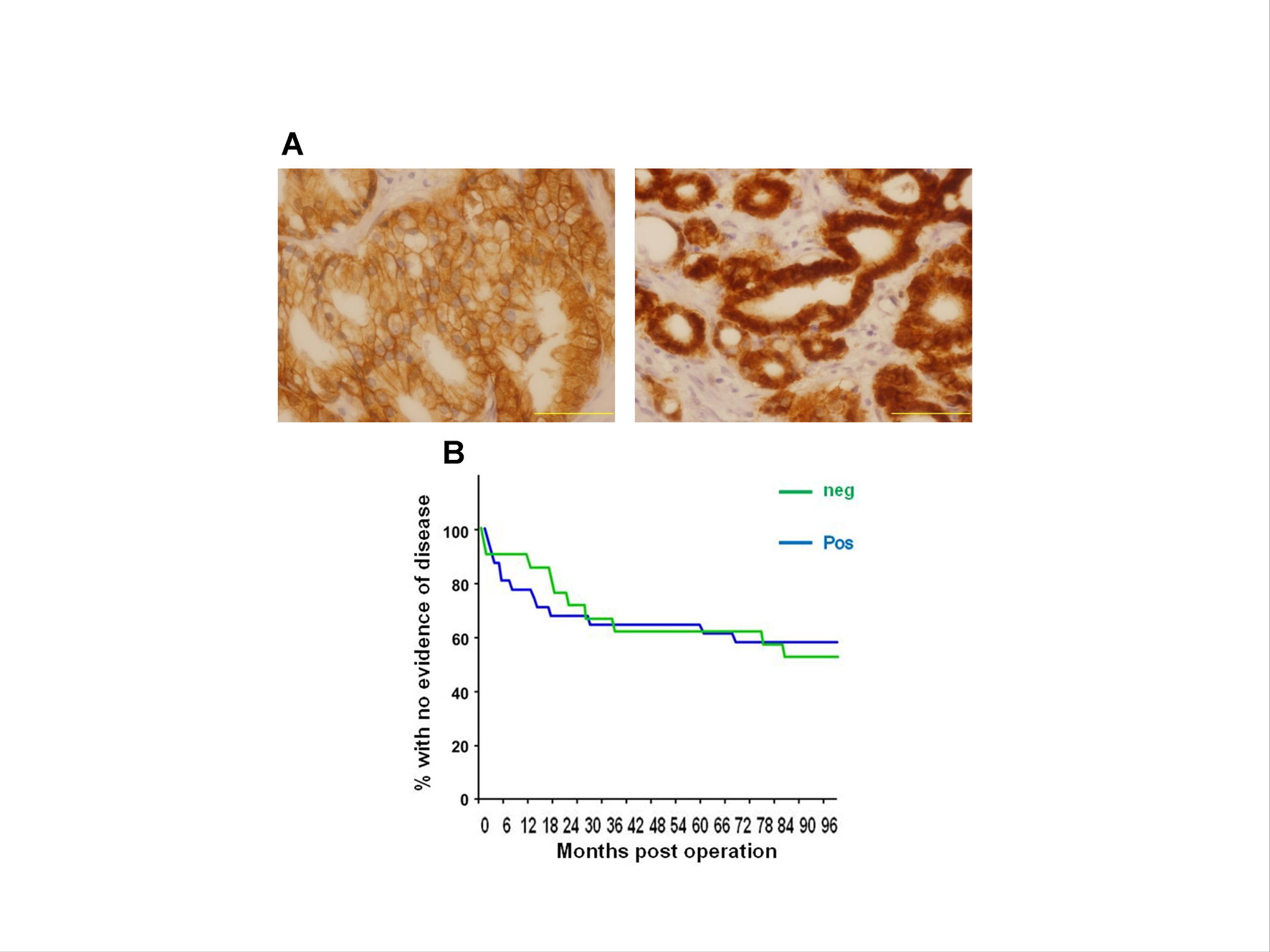

Supplement: Figure S1 — β-catenin activation does not correlate with prostate cancer patients' outcome. Primary prostate cancer tumors were immunostained using β-catenin antibodies. A. Representative photomicrographs of samples from negative (left) and positive (right) β-catenin stained tumors. B. Kaplan Meier curves plotting β-catenin positive (blue) vs. negative (green) patients' recurrence free interval. Scale bars in A, 50 µM. (1.02 MB TIF) [file pone.0009060.s001.tif]

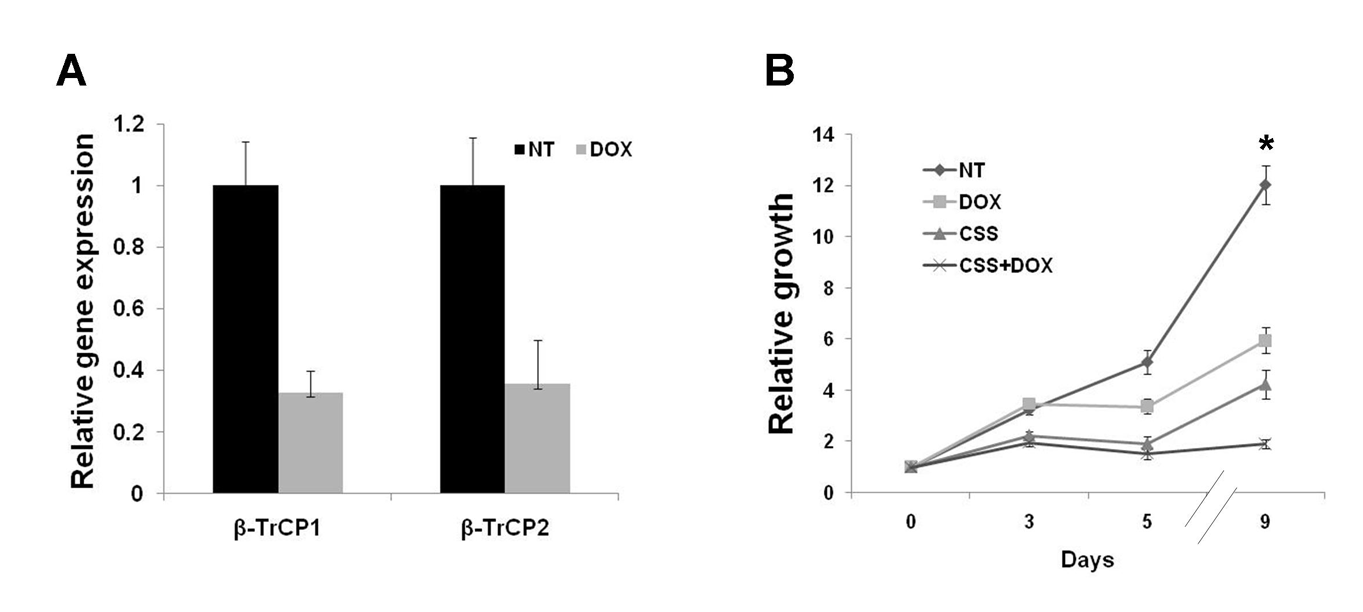

Supplement: Figure S2 — β-TrCP shRNA cooperates with androgen ablation to reduce LAPC4 cell growth. LAPC4 cell infected with the mentioned inducible lentiviral vector containing β-TrCP shRNA. A. qRT-PCR demonstrating efficient β-TrCP1 and β-TrCP2 knockdown. B. XTT assay was used to quantify cells proliferation rates (means ± S.E.M.). Error bars in A, SD. NT, no treatment; DOX, doxycycline; CSS, charcoal stripped serum. * All treatments were statistically different from control (p-value<0.05, t-test). (0.19 MB TIF) [file pone.0009060.s002.tif]

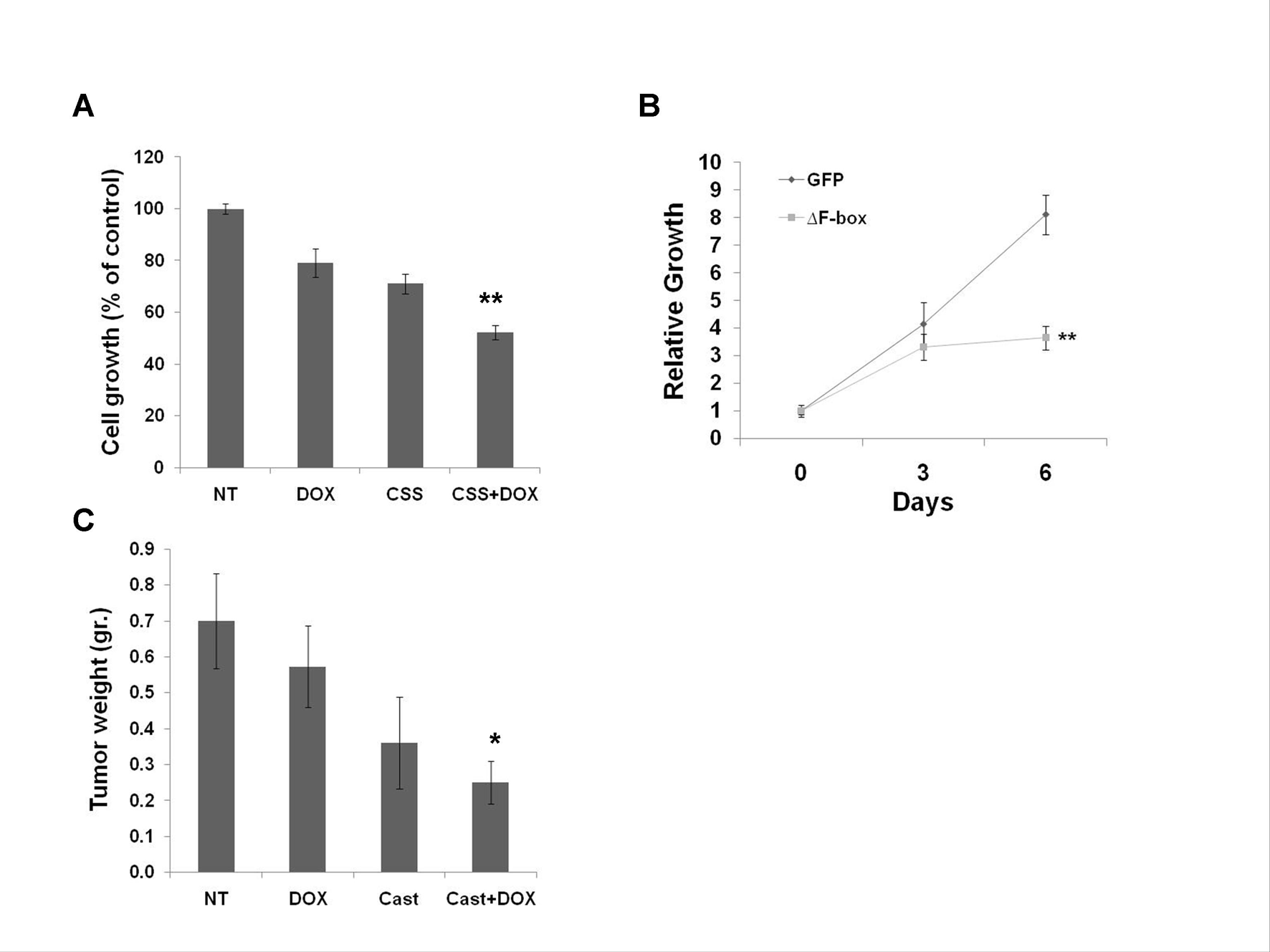

Supplement: Figure S3 — Dominant negative β-TrCP expression inhibits prostate cancer cell growth in vitro and in vivo. A. AT2.1 cells stably transfected with an inducible dominant negative β-TrCP were treated as indicated for 72 hours and subjected to MTT cell proliferation assay. B. LNCaP cells infected with lentiviral vector expressing eGFP (GFP) or dominant negative β-TrCP (ΔF-box) and assayed using the XTT reagent. C. Athymic 6–8 weeks male NUDE mice were divided into the 4 indicated groups (n≥4) and subcutaneously grafted with AT2.1 cells bearing the doxycycline dependent dominant negative β-TrCP construct. Tumor volumes were measured two weeks post injection. Shown are means ± S.E.M for A and C and means ± SD for B. * Significantly different from control group (p<0.05, t-test); ** Significantly different from all treatment groups (p<0.01, t-test). NT, no treatment; DOX, doxycycline; CSS, charcoal stripped serum; Cast, castrated mice. (0.20 MB TIF) [file pone.0009060.s003.tif]

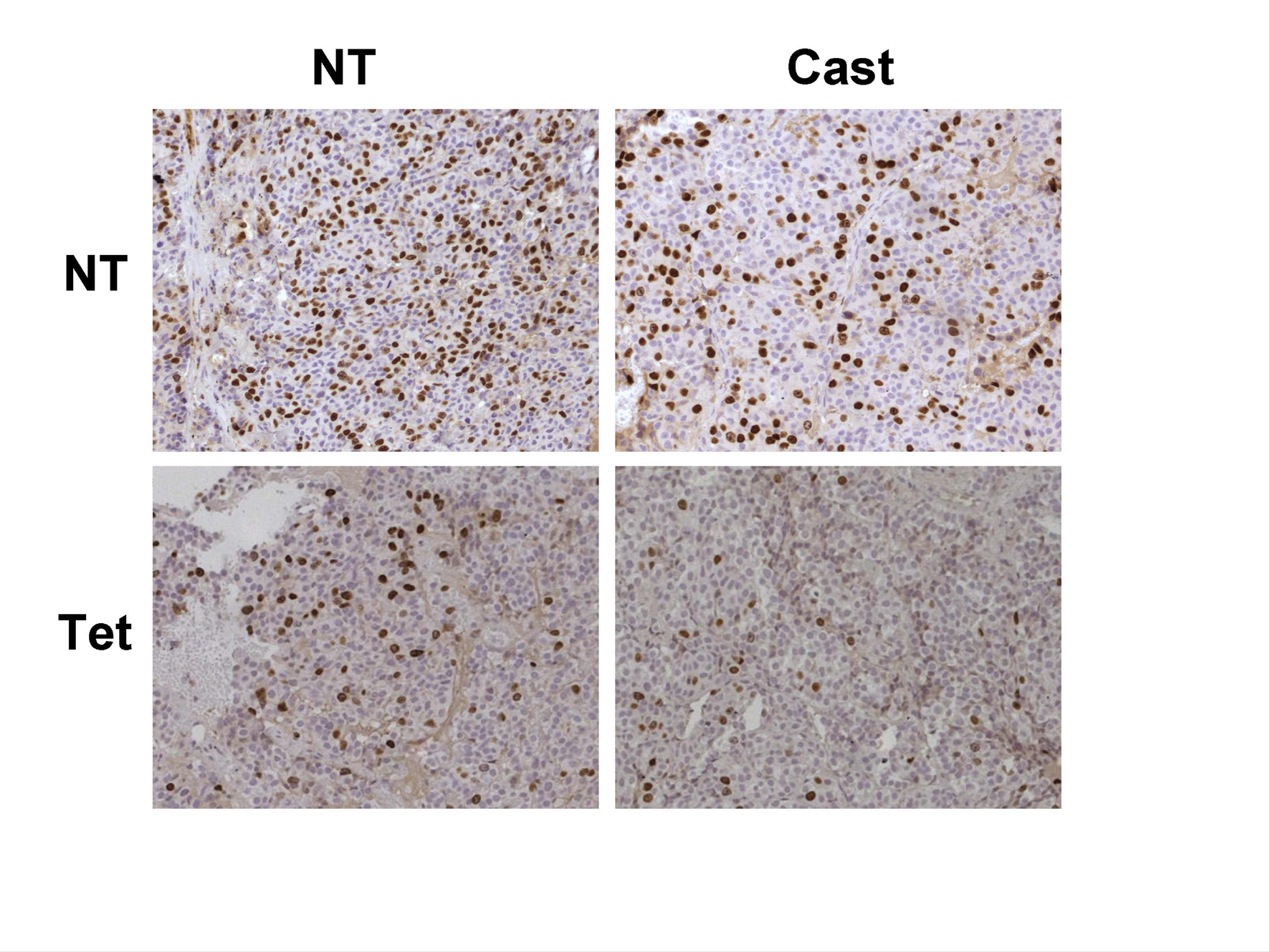

Supplement: Figure S4 — β-TrCP inhibition cooperates with androgen ablation treatment to reduce prostate cancer cells proliferation in vivo. LNCaP xenografts from treated Rag1−/− mice were immunostained with anti BrdU antibodies. Representative photomicrographs for each of the four treatment groups are shown. NT, no treatment; cast, castrated mice; Tet, tetracycline. (2.87 MB TIF) [file pone.0009060.s004.tif]

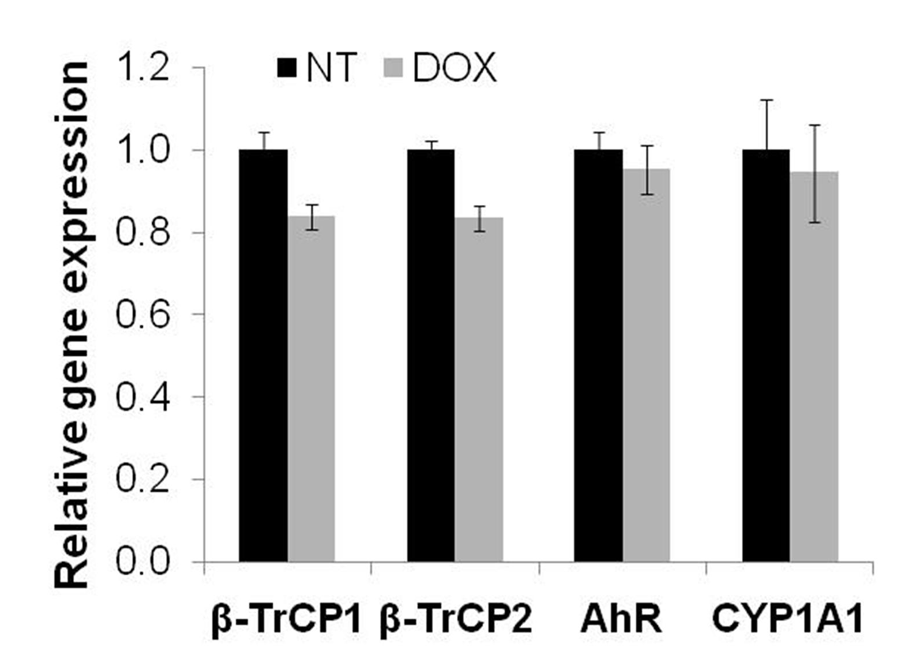

Supplement: Figure S5 — Doxycycline does not upregulate AhR. LNCaP cells infected with a GFP expressing lentiviral vector were subjected to qRT PCR analysis with the indicated primers. Means ± S.E.M of the relative genes expressions are shown. NT, no treatment; DOX, doxycycline. (0.12 MB TIF) [file pone.0009060.s005.tif]

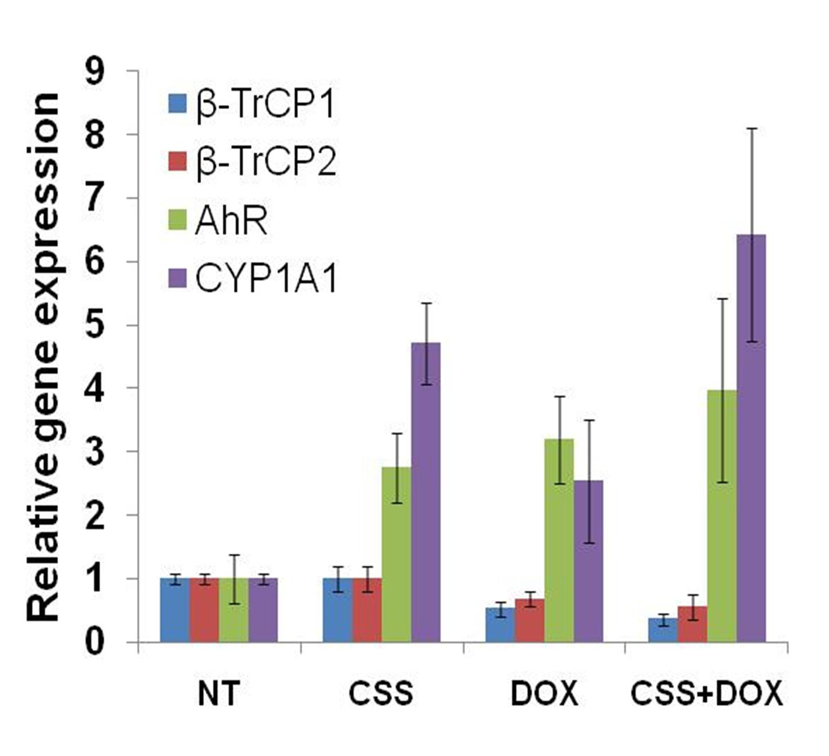

Supplement: Figure S6 — β-TrCP inhibition upregulates the AhR in LNCaP cells. LNCaP cells infected with an inducible shβ-TrCP lentiviral vector and treated as indicated were subjected to RNA extraction and qRT PCR analysis with the listed primers. CSS, charcoal stripped serum; DOX, doxycycline; Error bars, SD. (0.33 MB TIF) [file pone.0009060.s006.tif]

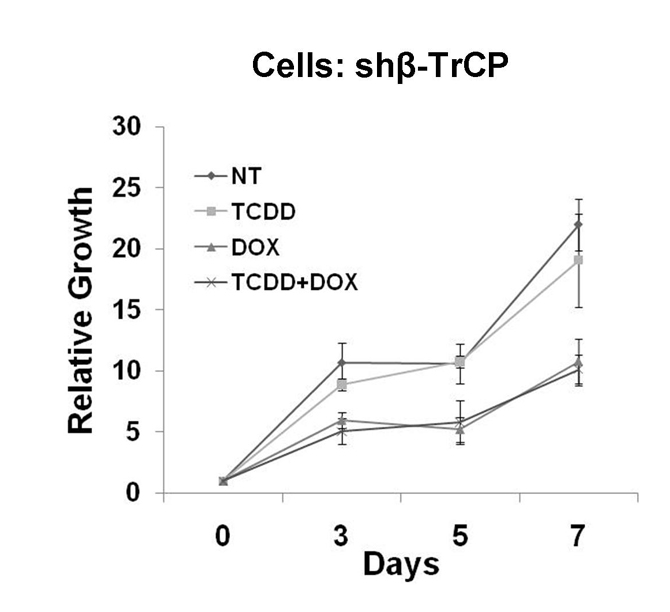

Supplement: Figure S7 — TCDD does not alter LNCaP cell growth in vitro. LNCaP cells were infected with a lentiviral vector harboring an inducible doxycycline dependent β-TrCP shRNA. Cells were treated with 1 µg/ml doxycycline, 10 nM TCDD or both and XTT assay was used to quantify cell growth at different time points. Shown are means ± SEM. NT, no treatment; DOX, doxycycline. (0.07 MB TIF) [file pone.0009060.s007.tif]
